# Protective eyewear for agricultural workers

**Published:** 2015

**Authors:** Ruchi Goel, KPS Malik

**Affiliations:** Ophthalmologist: Maulana Azad Medical College, New Delhi, India. **gruchi1@rediffmail.com**; Ophthalmologist: Subharti Medical College, Meerut, Uttar Pradesh, India. **malikkps@rediffmail.com**

**Figure 1.**
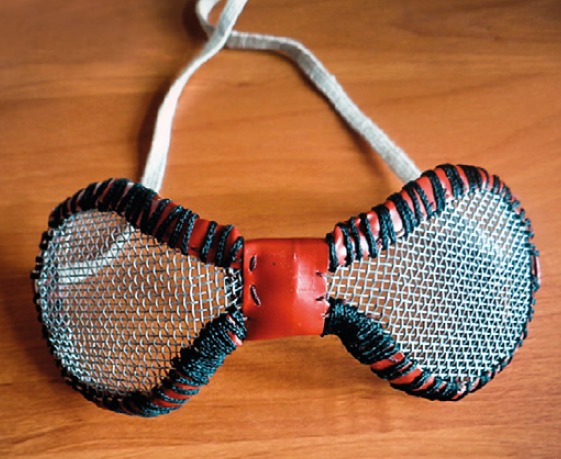
The locally made personal protective eyewear

Lids provide natural protection to the eyes. However, they may not provide sufficient protection in occupations such as agriculture, industrial work, certain sports, medicine, etc. Agricultural work is one of the riskiest occupations for the eye and protective eyewear can prevent eye injuries in 90% of cases.^1^

Despite the availability of a range of protective eyewear, the reasons for their non-use are perceived lack of protection, discomfort, undesirable appearance, interference with visual acuity, slowing down of work pace and no mandate from employers.^1^

To address the above problems and also issues like the eyewear falling off, fogging, limiting the field of vision, and cost, we came up with a design for protective eyewear which can be easily prepared locally at a minimal cost.

## Description

A 18 cm × 7 cm aluminium net used in windows is cut in the shape of goggles. The openings in the net are 2 × 2 mm in size. The edges are covered with a lining of thick cotton cloth and an elastic band is attached at the back. The nose band is covered with cloth as well. The eyewear is light and flexible and the net is placed about 15 mm anterior to the corneal surface.

We recommend the use of this inexpensive, home made device for agricultural workers. It meets all the requirements of ideal personal protective eyewear, apart from its inability to protect against UV radiation.
